# Emulsion stabilizing capacity of intact starch granules modified by heat treatment or octenyl succinic anhydride

**DOI:** 10.1002/fsn3.17

**Published:** 2013-02-07

**Authors:** Anna Timgren, Marilyn Rayner, Petr Dejmek, Diana Marku, Malin Sjöö

**Affiliations:** 1 Department of Food Technology Engineering and Nutrition, Lund UniversitySE 221 00, Lund, Sweden; 2 Speximo AB, Ideon Science Park, Scheelevägen 15SE 223 70, Lund, Sweden

**Keywords:** Granule size, heat treatment, OSA modification, Pickering emulsion, quinoa starch, starch granules

## Abstract

Starch granules are an interesting stabilizer candidate for food-grade Pickering emulsions. The stabilizing capacity of seven different intact starch granules for making oil-in-water emulsions has been the topic of this screening study. The starches were from quinoa; rice; maize; waxy varieties of rice, maize, and barley; and high-amylose maize. The starches were studied in their native state, heat treated, and modified by octenyl succinic anhydride (OSA). The effect of varying the continuous phase, both with and without salt in a phosphate buffer, was also studied. Quinoa, which had the smallest granule size, had the best capacity to stabilize oil drops, especially when the granules had been hydrophobically modified by heat treatment or by OSA. The average drop diameter (*d*_32_) in these emulsions varied from 270 to 50 μm, where decreasing drop size and less aggregation was promoted by high starch concentration and absence of salt in the system. Of all the starch varieties studied, quinoa had the best overall emulsifying capacity, and OSA modified quinoa starch in particular. Although the size of the drops was relatively large, the drops themselves were in many instances extremely stable. In the cases where the system could stabilize droplets, even when they were so large that they were visible to the naked eye, they remained stable and the measured droplet sizes after 2 years of storage were essentially unchanged from the initial droplet size. This somewhat surprising result has been attributed to the thickness of the adsorbed starch layer providing steric stabilization. The starch particle-stabilized Pickering emulsion systems studied in this work has potential practical application such as being suitable for encapsulation of ingredients in food and pharmaceutical products.

## Introduction

In an emulsion, drops need to be stabilized in order to avoid coalescence. Surfactants adsorbed to the interface of the two phases decrease the interfacial tension and may increase steric hindrance or electrostatic repulsion, which increases the stability of the emulsion. Proteins and surfactants are usually used as emulsifiers in food emulsions. However, polysaccharides have also been used to stabilize emulsions, especially gum arabic, modified celluloses, and starches (Dickinson 2009). When used as emulsion stabilizer, starch is usually gelatinized and/or dissolved (Tesch et al. 2002; Nilsson and Bergenståhl 2006), although recently also undissolved mechanically fractured starch has been used alone (Yusoff and Murray 2011) and in combination with proteins (Murray et al. 2011). Recently, even extremely small intact starch granules have been used to stabilize Pickering emulsions (Timgren et al. 2011; Rayner et al. 2012a).

Oil drops stabilized by dispersed particles, known as Pickering emulsions, were originally observed independently by Ramsden (1903) and Pickering (1907). Emulsions stabilized by solid particles are usually more stable against coalescence and Ostwald ripening compared with systems stabilized by surfactants (Binks 2002; Aveyard et al. 2003). The particles used are often of inorganic origin such as silica, fat crystals, proteins, or hydrocolloids (Dickinson 2010). The size of particles used for Pickering emulsions varies from nano to micron sized. The droplet size decreases with decreased particle size, but only as long as other properties, such as wettability, shape, and surface, are the same.

Starch granules naturally vary in size from 0.5 to 100 μm (Jane et al. 1994). Depending on botanical origin, the size distribution and shape of starch granules can differ substantially, as well as the ratio between the two starch polymers, amylopectin and amylose. Starch granules can exist in a variety of forms: smooth, rough, or edgy surface, and the shape can be spherical, ellipsoidal, flat-like discs, polygonal, or like rods (Jane et al. 1994).

Native starch is not hydrophobic, and thereby generally not suitable to adsorb to the interface of water and oil and thus to stabilize an emulsion. However, by modification of starches, the hydrophobicity can be increased. Starch can be chemically modified by treatment with different alkenyl succinic anhydrides, for example, octenyl succinic anhydride (OSA), which is approved for food applications at an added amount of up to 3% based on the dry weight of starch. The hydrophobic octenyl group and the carboxyl or sodium carboxylate group increased starches' ability to stabilize emulsions (Wurzburg 1995). Emulsification with gelatinized and dissolved OSA starch has been found to be independent of starch concentration (above necessary limit for stabilization), pH value, and ion valence when two varieties of Purity Gum (2000 and 539-E) were studied (Tesch et al. 2002). OSA starches from waxy corn and amaranth have also shown to have emulsification capacity, which was independent of the degree of substitution (DS) and of the two starch types studied (Bhosale and Singhal 2006). Previous studies on nondissolved starch (Yusoff and Murray 2011) showed that emulsions stabilized by OSA-modified starch granule fragments were stable for more than 3 months. Another way to increase the hydrophobicity of starch is by dry heating, causing starch granule surface proteins to change character from hydrophilic to hydrophobic (Seguchi 1984; Madivala et al. 2009). An advantage of thermal modification is that no specific labeling is required when used in food applications. Furthermore, the hydrophobic alteration is explicitly occurring at the granule surface.

In our preliminary experiments, oil-in-water emulsions stabilized by OSA-modified quinoa granules were stable for more than 2 months and could be tailored to be creaming, sinking, or buoyancy neutral, depending on the starch to oil ratio (Rayner et al. 2012b). Rheological properties and droplet size distributions have also been studied after an 8-week storage period (Marku et al. 2012).

The objective of this study was to investigate the emulsifying capacity and storage stability of a broad spectrum of starches in their granular form. The starches used were both native and hydrophobically modified and had different granule size and amylose/amylopectin composition. The impact of salt concentration on the emulsifying capacity was studied in order to simulate the conditions of different food systems. Furthermore, the starch concentration was varied to analyze the effects on emulsion droplet size. The results of this study will be used to identify starches suitable for further emulsification and encapsulation studies.

## Material and Methods

### Material

The following commercial starches have been investigated in this screening study: rice, waxy rice, maize, waxy maize, high-amylose maize (Hylon VII), and waxy barley (all from Lyckeby-Culinar AB, Sweden). Starch isolated from quinoa grains (Biofood-Biolivs AB, Sweden) by wet-milling (Rayner et al. 2012a) has also been included in the study. The starches have been studied in their native form, heat treated, and OSA modified. The continuous phase was a 5 mmol/L phosphate buffer with pH 7 with and without 0.2 mol/L NaCl, the dispersed phase was the medium-chain triglyceride oil Miglyol 812 (Sasol, Germany). OSA was donated by Lyckeby-Culinar AB.

### Methods

#### Hydrophobic treatment by OSA modification of starch granules

Starch was thoroughly suspended in the double part by weight of water using a stainless-steel propeller and the pH was adjusted to 7.8. Four equal amounts of OSA (totally 4% based on weight of starch) were added with an interval of 15 min and the pH was maintained at 7.4–7.9 by adding 1 mol/L NaOH solution drop by drop. When the pH was stable for at least 15 min, the starch solution was centrifuged at 3000*g* for 10 min and washed twice with water and once with citric acid solution (pH 4.5) before the starch was air dried at room temperature for at least 48 h.

The OSA substitution was determined by a titration method used in the industry (Lyckeby-Culinar AB). Briefly, 5 g (dry weight) of starch was dispersed in 50 mL 0.1 mol/L HCl and stirred for 30 min. The slurry was centrifuged at 3000*g* for 10 min, washed once with 50 mL ethanol (90%) and twice with water before the starch was suspended in 300 mL water, cooked in a boiling water bath for 10 min, and cooled to 25°C. The starch solution was titrated with 0.1 mol/L NaOH to pH 8.3. A blank was simultaneously titrated with native starch of the same origin as the OSA starch as a sample. The percentage of carboxyl groups from OSA on the starch granules was calculated by


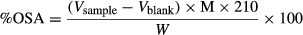
(1)

where V is the volume (mL) of NaOH required for the sample and the blank titration, M is the molarity of NaOH (0.1 mol/L), W is the dry weight (mg) of the starch, and 210 is the molecular weight of octenyl succinate group.

#### Hydrophobic modification by heat treatment of starch granules

Dry starch was placed on glass dishes and heat treated in an oven at 120°C for 150 min in order to increase the hydrophobicity of starch granules and thereby achieve a higher affinity for a hydrophobic interface. This type of heat modification is based on work by Seguchi (1984) where several time–temperature combinations were considered and our preliminary study on heat-treating quinoa starch granules (Rayner et al. 2012a,2012b).

#### Particle size measurements of starch granules

The particle size distributions of the starch were measured using laser diffraction (Coulter LS130, Beckman Coulter, U.K.) in a flow through cell. The optical mode was Mie with a refractive index (RI) of starch of 1.54 (Bromley and Hopkinson 2002). A small volume of starch granules was suspended in water and added to the flow system that pumped the samples through the optical chamber for measurements.

#### Imaging of starch granules

The starch granule samples were sputter coated with gold and images recorded in a scanning electron microscope (FegSEM, JEOL model JSM-6700F, Japan) operated at 5 kV and a working distance at 8 mm. The lower electron imaging (LEI) detection mode was used as LEI gives a clear three-dimensional image of the surface of the samples studied. The LEI detector combines signals from both secondary electrons and of backscattered electrons.

#### Preparation of starch granule-stabilized emulsions

Emulsions were prepared in glass test tubes with 4 mL of the continuous phase, 2 mL of the oil phase, and 100–400 mg starch (50–200 mg/mL oil) by mixing with an Ystral (D-79282, Ballrechten-Dottingen, Germany) at 11,000 rpm for 30 sec. Approximately 1 mg of the oil-soluble dye Solvent Red 26 was added to the top of the emulsions after 24 h, and the test tubes were gently turned three times 1 h after addition in order to distribute the color in the entire sample. The color change in the emulsion was observed. The emulsions were shaken with a vortex mixer (VM20, Chiltern Scientific Instrumentation Ltd., U.K.) for 5 sec after another 2 h to promote the interaction of the dyed oil with the rest of the emulsion drops. The color after vortex is a measure of the orthokinetic stability of the formed drops. Stable drops do not have an exchange with the lipophilic dye; hence, the emulsion phase will remain white. An increased red-colored emulsion phase indicates that the drops were less stabilized by the adsorbing starch granules or there is a free oil phase in the system. Afterward, the samples were stored at room temperature for 6 days before further analysis (described below). The samples were stored for another period of 2 years in sealed test tubes at room temperature before reanalyzing.

#### Light microscopy of starch granule-stabilized emulsions

For microscopy of the emulsions, an Olympus BX50 (Japan) microscope with Plan 2×, UMPlanFL 5× and 10×, and LMPlanFL 20× and 50× objectives (Olympus) was used. The light was transmitted using a polarization filter (U-ANT, Olympus) and a wavelength filter (U-TP530, Olympus). The emulsion was diluted five times with buffer corresponding to the continuous phase of the sample. The samples were placed on a microscopic slide, and the drops were studied immediately without cover glass. Images of the drops were taken with a 1280 × 960 resolution camera (DFK 41AF02, The Imagingsource, Germany) connected to the microscope, and the images were processed using the Java image processing program ImageJ (version 1.42m).

#### Analysis of starch granule-stabilized emulsions after first storage period

The test tubes with the emulsified samples were photographed 1 h, 24 h, and 6 days after the vortex treatment, and the images of the samples were analyzed in ImageJ. The emulsifying capacity of the starches and the stability of the emulsions were expressed as the volume of the emulsion to the total volume of the sample, often referred to as the emulsification index (EI). The amount of material, generally remaining starch, at the bottom of the test tube was also calculated. The EI (McClements 2007) was calculated as follows:


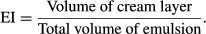
(2)

The drop size distribution of the emulsions was determined from microscopic images. The diameter of at least 250 drops was measured with ImageJ in the samples that contained drops that had a diameter smaller than 1.4 mm. The surface mean drop diameter (*d*_32_) and the volume mean diameter (*d*_43_) were calculated using equations (3) and (4):


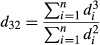
(3)


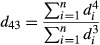
(4)

where *d* is the measured diameter of a drop and n is the total number counted. The coefficient of variation (CV) as percentage and the standard deviation have been calculated according to the following equations to arrive at the distribution of the emulsion drops in each sample.


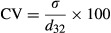
(5)


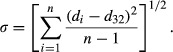
(6)

#### Analysis of starch granule-stabilized emulsions after a 2-year storage period

After an additional 2 years of storage at room temperature (22°C) in glass test tubes, the starch granule-stabilized emulsions were photographed as described above. The starch granule-stabilized emulsions which had an initial mean particle size less than 120 μm determined by equation (4) were subsequently remeasured using light scattering. This cutoff was chosen as droplets significantly larger than 150 μm can be broken into smaller drops during measurement. A laser diffraction particle size analyzer (Mastersizer 2000 Ver.5.60, Malvern, Worcestershire, U.K.) was used in order to determine the particle size distribution of the starch granule-stabilized emulsion oil drops. The sample was added to the flow system containing milliQ-water and was pumped through the optical chamber at a pump velocity of 2000 rpm. The RI of the sample was set to 1.54 as starch (Bromley and Hopkinson 2002), the RI of the continuous phase was set to 1.33 (water), and the obscuration was between 10% and 20%.

## Results and Discussion

### Starches

**Figure 1:**
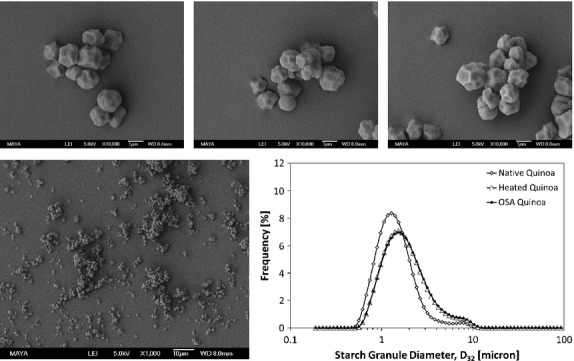
Scanning electron micrographs (top and left) and *d*_32_ size distributions (right) (Coulter LS130) of starch granules. Top: Native, heat-treated, and octenyl succinic anhydride (OSA)-modified quinoa (10,000×), left: OSA quinoa (1000×).

**Figure 2:**
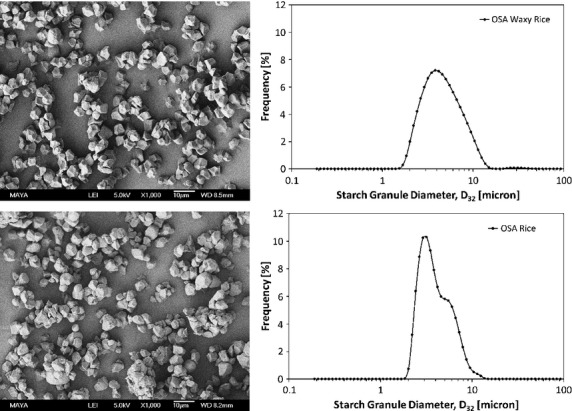
Scanning electron micrographs (left) and *d*_32_ size distributions (right) (Coulter LS130) of starch granules. Top-left: Octenyl succinic anhydride (OSA) waxy rice (1000×), bottom-left: OSA rice (1000×).

**Figure 3:**
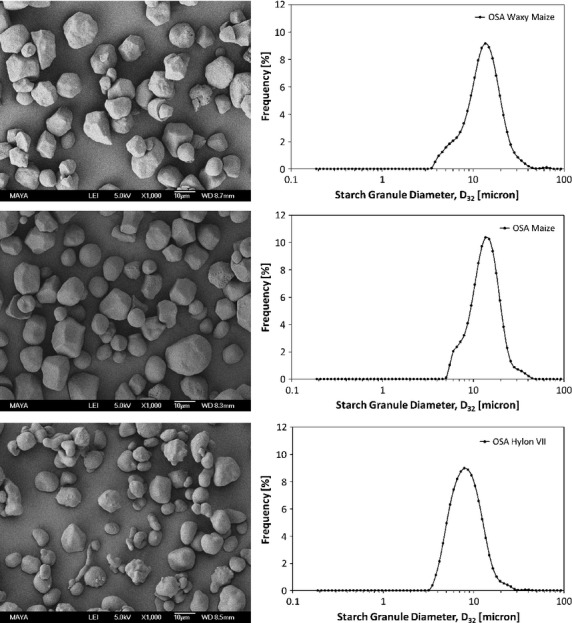
Scanning electron micrographs (left) and *d*_32_ size distributions (right) (Coulter LS130) of starch granules. Top-left: Octenyl succinic anhydride (OSA) waxy maize (1000×), middle-left: OSA maize (1000×), bottom-left: OSA high-amylose maize (1000×).

**Figure 4:**
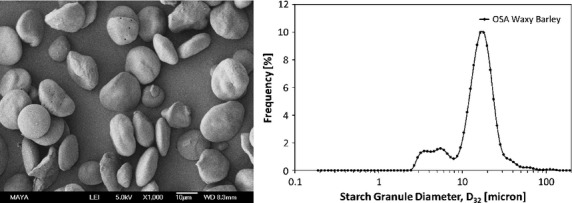
Scanning electron micrograph (1000×) (left) and *d*_32_ size distributions (right) (Coulter LS130) of octenyl succinic anhydride (OSA) waxy barley starch granules right.

The starches selected for this study had different granule size, with quinoa as the smallest one followed by rice, maize, and barley, and these granules also had different shapes observed in SEM images in Figures 1–4. Quinoa granules had a *d*_32_ of approximately 2 μm and had smooth edges (Fig. 1), while rice had sharp-edged granules with a *d*_32_ of 4.5 and 5.4 μm for waxy and normal rice, respectively (Fig. 2). Waxy and normal maize had both smooth and sharp edges of their granules, whereas the high-amylose maize (Hylon VII) was smoother and also had some rod-shaped granules (Fig. 3). The mean size of the maize granules was 9.3 μm for high-amylose maize and 15 μm for the other two maize varieties. Barley starch granules were smoothly shaped spheres and oblate spheroids with a mean *d*_32_ of 17 μm (Fig. 4), whereas quinoa, rice, and maize had more irregular polygonal shapes. Figure 1A shows the effect of treatment on the quinoa starch granules. The shape of the granules was similar for all three samples, but the size was increased for the granules that had been subjected to heat treatment or OSA modification, which partly was due to a higher degree of aggregation caused by the increased hydrophobicity. Individual quinoa starch granules had a size between 0.7 and 2.2 μm.

Starch has a natural variation in amylose/amylopectin composition and the normal varieties have an amylose content of around 20–30%. Waxy starches have a very low content of amylose, and in this study, waxy varieties of rice, barley, and maize were used. A variety of maize with a high content of amylose (Hylon VII) with 70% amylose was also used in order to see the emulsification behavior in a larger spectrum of the amylose content. It has been shown (Shogren et al. 2000) that OSA binding is nonuniform at molecular scale and affected by differences in starch molecular branching.

**Figure 5:**
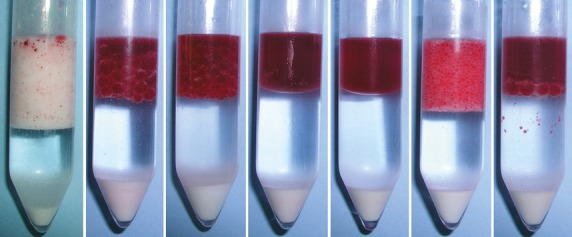
Native starches as drop stabilizer, conditions: 100 mg starch/mL oil and 0.2 mol/L NaCl. The samples correspond to sample ID #'s in Table 1, from left to right, #5 (quinoa), #11 (waxy rice), #17 (rice), #22 (waxy maize), #28 (maize), #33 (Hylon VII), and #39 (waxy barley).

The color of the dyed emulsions is a measure of the interaction of the oil in the formed drops as the dye was added on top of the samples after the emulsification and before the samples were mixed in a vortex. Stable drops did not have an exchange with any dyed oil; hence, the emulsion phase remained white. An increased red-colored emulsion phase indicated that the drops were less stabilized by the adsorbing starch granules and that the interface barrier was more sparse, which lead to exchange of oil between the drops. The size of the drops correlated with the color and stability of the emulsion, that is, the more red the sample appeared the larger (or lack of) droplets there were in the system. This was mainly dependent on the size of the stabilizing granules, but also the shape of the granules had an impact. Quinoa, which had a unimodal drop size distribution of small granules (*d*_32_ = 2 μm), had the preeminent best capacity to stabilize an emulsion at the circumstances used in this study. Native starch granules are supposed to be inefficient as oil drop stabilizers due to the low hydrophobicity; however, native quinoa and to some extent Hylon VII were able to stabilize the formed emulsion drops as seen in Figure 5, which was a somewhat unexpected result.

### Effect of hydrophobic treatments on the starches' emulsifying capacity

All starches in this study have been used in their native (Table 1), heat-treated, and OSA-modified forms (Table 2). Seguchi (1984) suggested starch surface protein to cause the hydrophobic effect of heat-treated starch granules. All starch granules have some proteins bound to their surface, and for the small quinoa granules, the total large surface of all the granules may give enough hydrophobicity to stabilize drops, even though the drops stabilized by native quinoa starch were larger than when the modified (heat or OSA) quinoa starches were used.

**Table 1:** Native starch (100 mg starch/mL oil) of different origin mixed with oil and phosphate buffer with pH 7 and 0.2 mol/L NaCl

Starch origin	Sample ID	Emulsification index (6 days after vortex)	Sediment fraction^1^ (mm^3^/mg)	Drop size (6 days after vortex)		

				*d*_32_ (μm)	*d*_43_ (μm)	CV (%)
Quinoa	5	0.60	0.35	320	370	46
Waxy rice	11	0.40	2.3	>1 mm	>1 mm	–
Rice	17	0.42	1.7	>1 mm	>1 mm	–
Waxy maize	22	0.38	1.5	No drops	No drops	–
Maize	28	0.38	1.2	No drops	No drops	–
Hylon VII	33	0.48	1.2	980	>1 mm	51
Waxy barley	39	0.38	1.3	>1 mm	>1 mm	–

CV, coefficient of variation.

1 Ratio of sediment volume to added starch.

**Table 2:** Different treatment of the starch. Starch (100 mg starch/mL oil) mixed with oil and phosphate buffer with pH 7 and 0.2 mol/L NaCl

Starch origin	Treatment of the starch	Sample ID	Emulsification index(6 days after vortex)	Sediment fraction^1^ (mm^3^/mg)	Drop size (6 days after vortex)		

					*d*_32_ (μm)	*d*_43_ (μm)	CV (%)
Quinoa	Heat treated	6	0.68	0.31	160	170	41
Quinoa	OSA 1.8%	7	0.78	0.015	76	79	40
Quinoa	OSA 2.9%	9	0.77/0.74^2^	0.03/0.02^2^	100/110^2^	110/120^2^	37/37^2^
Waxy rice	Heat treated	12	0.44	2.0	>1 mm	>1 mm	–
Waxy rice	OSA 3.8%	13	0.59	0.55	440	500	42
Rice	Heat treated	18	0.46	1.7	530	590	71
Rice	OSA 2.8%	20	0.55/0.62^2^	0.70/0.33^2^	530/350^2^	560/440^2^	75/63^2^
Waxy maize	Heat treated	23	0.39	1.9	No drops	No drops	–
Waxy maize	OSA 3.3%	24	0.64	0.15	500	540	38
Maize	Heat treated	29	0.38	1.5	No drops	No drops	–
Maize	OSA 2.6%	31	0.50/0.59^2^	0.65/0.14^2^	1300/720^2^	1400/750^2^	30/29^2^
Hylon VII	Heat treated	34	0.52	1.1	830	880	40
Hylon VII	OSA 3.1%	35	0.54	0.90	710	750	27
Waxy barley	Heat treated	40	0.50	0.90	890	930	41
Waxy barley	OSA 3.6%	42	0.58/0.60^2^	0.27/0.22^2^	690/670^2^	720/700^2^	27/27^2^

OSA, octenyl succinic anhydride; CV, coefficient of variation.

1 Ratio of sediment volume to added starch.

2 Replicate results from two different samples.

For the purpose of comparison among the starch varieties studied, we have quantified the emulsifying capacity of the starch granules via mean diameter (*d*_43_) of the stabilized oil drops and the emulsifying index at equal starch to oil ratios. Quinoa had definitely best emulsifying capacity followed by rice, which only had slightly larger granule size, but the granules were more irregularly shaped with sharp edges. The emulsifying capacity was similar for the two varieties of OSA-modified rice (Table 2). Waxy and normal maize had also irregularly shaped granules, which can be one reason to the slightly less stabilizing capacity of maize compared with barley that had larger granule size but on the other hand smoother shaped granules. A reduced surface contact of large particles due to surface roughness or sharp edges seemed to have a negative impact on the emulsifying power (Vignati et al. 2003). Another reason was probably the bimodal size distribution of barley (Fig. 4) where the smaller granules potentially increased the drop stability and decreased the drop size. Four samples were produced in duplicate: quinoa, rice, maize, and waxy barley, all with 100 mg OSA starch/mL oil and buffer with salt as the continuous phase (Table 2). Quinoa and waxy barley, which produced stable emulsions, showed good reproducibility regarding drop size, sediment fraction, and emulsifying index, whereas the reproducibility of the results for rice and maize was poor.

The stabilizing capacity of waxy and normal maize was similar (Table 2), but the maize with a high content of amylose (Hylon VII) showed a different pattern. The three Hylon VII samples in native in Table 1 and modified in Table 2 had only minor disparities in emulsion fraction and drop size regardless of the treatment of the starch granules. The rod-shaped granules, which were about 10% of all the granules, seemed to have a large impact on the overall stabilizing capacity. It has been previously shown (Madivala et al. 2009) that long particles with an aspect ratio over 4 are more effective emulsifier than less elongated particles of similar wettability, which could explain this result.

**Figure 6:**
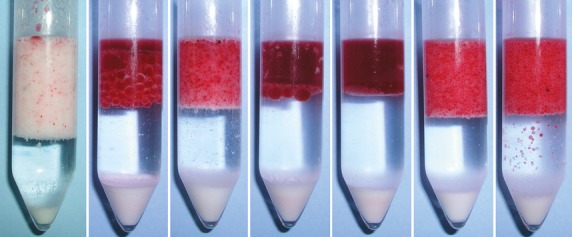
Heat-treated starches as drop stabilizer, conditions: 100 mg starch/mL oil and 0.2 mol/L NaCl. The samples correspond to sample #'s in Table 2, from left to right, #6 (quinoa), #12 (waxy rice), #18 (rice), #23 (waxy maize), #29 (maize), #34 (Hylon VII), and #40 (waxy barley).

The heat-treated starches (Fig. 6) were somewhat better stabilizers than the native starches (Fig. 5) as the hydrophobicity of the surface proteins should have increased as suggested by Seguchi (1984)). Especially, the drops stabilized by quinoa, rice, and waxy barley had a decreased drop size (refer to Tables 1 and 2). The hydrophobicity of the starch granules had apparently increased, but not sufficiently enough so that all types' granules were able to act as stabilizers. Quinoa to the greatest extent, as well as high-amylose maize (Hylon VII) and waxy barley to a lesser extent, could stabilize emulsion drops after heat treatment. However, the OSA modification was significantly more efficient for emulsion stabilization (as described below). Therefore, the heat treatment process was not further evaluated.

**Figure 7:**
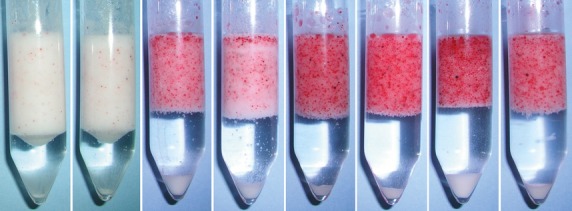
Octenyl succinic anhydride (OSA)-modified starches as drop stabilizer, conditions: 100 mg starch/mL oil and 0.2 mol/L NaCl. The samples correspond to sample #'s in Table 2, from left to right, #7 (quinoa OSA 1.8%), #9 (quinoa OSA 2.9%), #13 (waxy rice), #20 (rice), #24 (waxy maize), #31 (maize), #35 (Hylon VII), and #42 (waxy barley).

The OSA-modified starches were all able to stabilize oil drops (Fig. 7 and 2), but the utilization of the granules was not complete as free starch to varying degrees existed as sediment in the test tubes, and is discussed further in the *Starch concentration* section below. The content of OSA was between 2.6% and 3.6% for all starches, with the exception of quinoa which was also modified to a lower degree of 1.8%. No differences could be seen between the quinoa samples with the two degrees of OSA regarding drop size, volume fraction of emulsion, or stability. Even though the OSA has been shown to be distributed both in the interior and on the surface of the granule (Shogren et al. 2000), our results indicated that the OSA binding of 1.8% gave enough granule surface hydrophobicity to stabilize an emulsion. Starch modified with 3% OSA is commercially available and approved as a food additive.

### Storage stability

**Figure 8:**
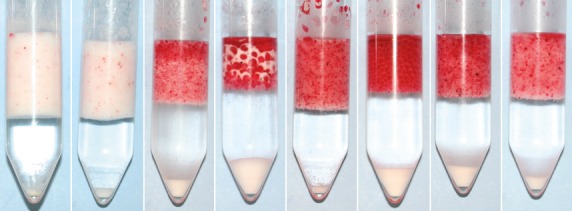
Octenyl succinic anhydride (OSA)-modified starches as drop stabilizer after 2 years of storage, conditions: 100 mg starch/mL oil and 0.2 mol/L NaCl. The samples correspond to sample #'s in Table 2, from left to right, #7 (quinoa OSA 1.8%), #9 (quinoa OSA 2.9%), #13 (waxy rice), #20 (rice), #24 (waxy maize), #31 (maize), #35 (Hylon VII), and #42 (waxy barley).

**Figure 9:**
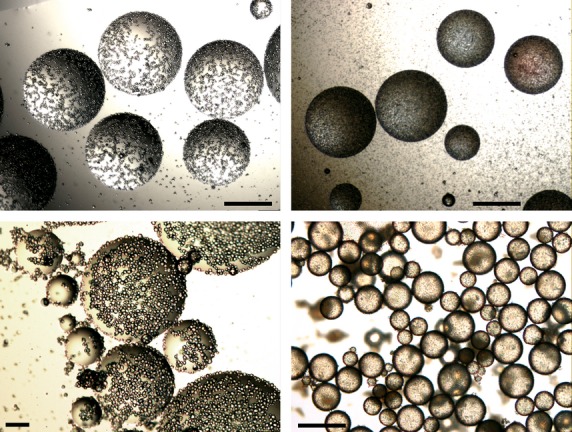
Octenyl succinic anhydride (OSA)-modified starches, 200 mg starch/mL oil, 0.2 mol/L NaCl after 2 years of storage. Top-left: waxy barley (20×, scale bar 500 μm), top right: rice (20×, scale bar 500 μm), bottom-left: maize (50×, scale bar 100 μm), bottom-right: quinoa (100×, scale bar 100 μm).

After storage at room temperature for a period of 2 years, the starch granule-stabilized emulsions were reexamined to see if they remain stable. Visual inspection of the test tubes showed that in all cases where there had been successful initial droplet stabilization, that is, in all the OSA-treated starches and some of the heat-treated starches, droplets remained and the emulsion layer had remained (Fig. 8). This was even the case for very large droplets that were visible to the naked eye. Figure 9 shows the micrographs of stored emulsions stabilized by OSA-modified starches (waxy barley, rice, maize, and quinoa) at 200 mg starch/mL oil. In these images, the larger granules of waxy barley and maize are clearly seen on the drop surface but with significant gaps between them. We would have expected a higher degree of coverage to necessary for these drops to have been formed at all, and especially not after 2 year of storage. However, Pickering emulsions have been shown to be adequately stabilized even when silica (0.5–0.8 μm) (Vignati et al. 2003) or spores particles (∼25 μm) (Binks et al. 2005) were highly unevenly distributed at the surface of the drops. In samples that had an initial mean droplet size less than 120 μm, in this case, the OSA quinoa starch stabilized emulsions; the particle size distribution was measured by light scattering, and the mean *d*_43_ as well as the mode (peak value of *d*_43_) is reported in Table Table 3. After 2 years of storage, the mean droplet diameter had not changed significantly, ±3% of initial, which is within the measurement error. This surprising stability of comparatively large emulsion drops (50–100 μm) over a range of 2 years has been supported by observations from earlier work. In previous studies with OSA-modified quinoa, we have detected no change in mean particle size distribution over a period of 2 months with various volume fractions of oil 12.5–33.3% having 214 mg starch/mL oil (Timgren et al. 2011).

**Table 3:** Changes in mean drop size after 2 years of storage for OSA-modified quinoa starch granule-stabilized emulsions

Sample ID#	OSA level	Continuous phase	Starch (mg/mL)	6 days, *d*_43_ (μm)	2 years	

					*d*_43_ (μm)	Mode *d*_43_ (μm)
3	OSA 1.8%	No salt	100	81	82	83
4	OSA 2.9%	No salt	100	87	84	83
7	OSA 1.8%	0.2 mol/L NaCl	100	79	76	81
9	OSA 2.9%	0.2 mol/L NaCl	100	110/120^1^	93/96^1^	97/101^1^
10	OSA 2.9%	0.2 mol/L NaCl	200	55	52	54

OSA, octenyl succinic anhydride.

1 Replicate results from two different samples.

### Effect of continuous phase salt content on emulsion properties

**Figure 10:**
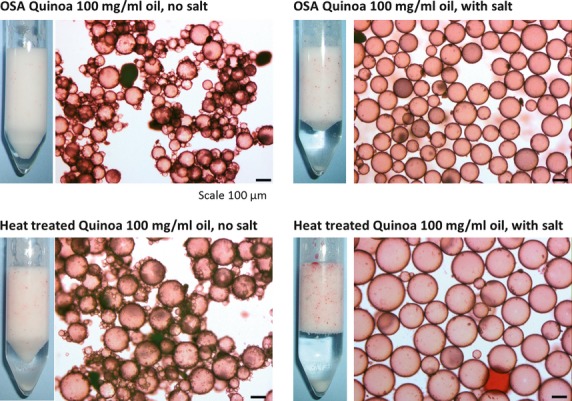
Samples and microscopic images of emulsions 6 days after vortex. Top: Octenyl succinic anhydride (OSA)-modified quinoa 100 mg starch/mL oil, and continuous phase without (left) and with (right) salt 0.2 mol/L NaCl. Bottom: Heat-treated quinoa 100 mg starch/mL oil, and continuous phase without (left) and with (right) salt 0.2 mol/L NaCl.

Two different phosphate buffers, with and without 0.2 mol/L NaCl, were used as continuous phase, and the pH was 7 in both buffers (Table 4). Preliminary tests (not shown) indicated only marginal variations at different pH (4–7) and salt concentrations (0.1–0.4 mol/L NaCl) as the main stabilizing mechanism of the starch granules is steric repulsion, which is insensitive to changes in pH. However, the difference in drop formation pattern was considerable between buffers with or without salt. The difference was apparent on both macro- and microscopic levels for the hydrophobically modified starch granules but not for the native granules. The samples with heat-treated or OSA-modified quinoa are shown in Figure 10.

**Table 4:** OSA-modified starch (100 mg starch/mL oil) mixed with oil and phosphate buffer (pH 7) without salt and with 0.2 mol/L NaCl

Starch origin	Continuous phase (salt concentration)	Sample ID	Emulsification index (6 days after vortex)	Sediment fraction^1^ (mm^3^/mg)	Drop size (6 days after vortex)		

					*d*_32_ (μm)	*d*_43_ (μm)	CV (%)
Quinoa	No salt	4	0.94	0	74	87	77
Quinoa	0.2 mol/L NaCl	9	0.77/0.74^2^	0.03/0.02^2^	100/110^2^	110/120^2^	37/37^2^
Rice	No salt	16	0.75	0.12	100	170	70
Rice	0.2 mol/L NaCl	20	0.55/0.62^2^	0.70/0.33^2^	530/350^2^	560/440^2^	75/63^2^
Maize	No salt	27	0.69	1.0	420	470	57
Maize	0.2 mol/L NaCl	31	0.50/0.59^2^	0.65/0.14^2^	1300/720^2^	1400/750^2^	30/29^2^
Waxy barley	No salt	38	0.76	0.040	370	460	65
Waxy barley	0.2 mol/L NaCl	42	0.58/0.60^2^	0.27/0.22^2^	690/670^2^	720/700^2^	27/27^2^

OSA, octenyl succinic anhydride; CV, coefficient of variation.

1 Ratio of sediment volume to added starch.

2 Replicate results from two different samples.

When a continuous phase without salt was used, the emulsions had a distinct cone shape formed by the tip of test tubes during emulsification and remained during creaming (left images in Fig. 10). This phenomenon indicated a weakly aggregated emulsion layer with a yield stress. However, this shape was less obvious in the presence of salt (right images in Fig. 10). In addition, the volume fraction of the emulsion was larger, and the starch sediment was less in the systems without salt. The drop size distribution also had a different character where the emulsions without salt had bimodal drop size distributions with a larger CV and a smaller *d*_43_ than the drops in the salt-containing emulsions, which had a more unimodal distribution. These observations can to a large extent be explained by the appearance of the drop systems shown in the microscopic images in Figure 6. In the absence of salt (left micrographs in Fig. 10), the emulsion drops formed a more rigid open network of large drops, small drops, and starch granule clusters, whereas in the systems with salt (right micrographs in Fig. 10), the drops were less efficiently stabilized (as evidenced by the presence of dye containing areas) and coalesced to a uniform, larger size without significant aggregation. Almost exactly the same behavior, with uniform droplets at higher ionic strength and varying sizes and aggregation at low ionic strength, has been reported for hexadecane in water emulsions stabilized with silica particles (Gautier et al. 2007). The effect was found to be reversible, and it was proposed that it was caused by a dipole force which is always attractive between partially wetted particles anchored at two opposing particle interfaces at low ionic strength.

### Starch concentration

**Figure 11:**
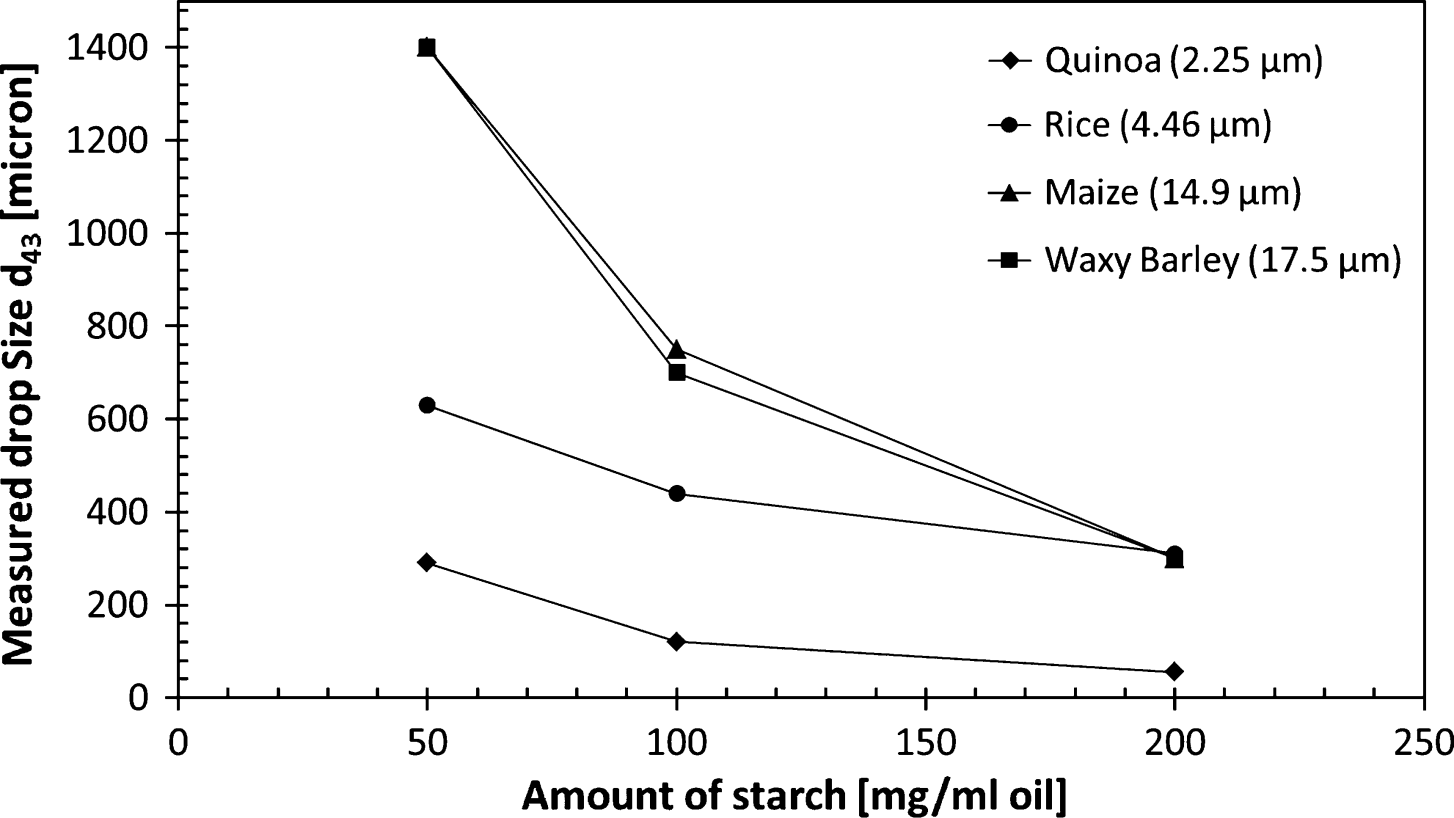
Drop size as a function of amount of added starch for four varieties of starch: quinoa, rice, maize, and waxy barley, all of which were octenyl succinic anhydride (OSA) modified and in a 0.2 mol/L NaCl phosphate buffer. Amount of added starch corresponds to 1.1, 2.2, and 3.9 vol% of the total system (i.e., 50, 100, and 200 mg/mL oil).

The effect of starch concentration on emulsification was studied on four varieties of starch: quinoa, rice, maize, and waxy barley, all of which were OSA modified and used in a 0.2 mol/L NaCl phosphate buffer (Table 5). These conditions were used as they had the best emulsification result in initial screening studies; the emulsions with salt had more uniform drop size distributions and less network formation. The mass of added starch was 100, 200, and 400 mg (50, 100, and 200 mg starch/mL oil), which corresponds to approximately 3.2, 6.2, and 11.8 vol% of the oil (or 1.1, 2.2, and 3.9 vol% of the total system), respectively. The measured drop size (*d*_43_) versus amount of added starch is plotted in Figure 11 for quinoa, rice, maize, and waxy barley. The drop size decreased with increased starch concentration and the drops were smaller for starches with small granule size at the same concentration. It has been previously shown that the average drop size of emulsions stabilized by solid particles decreases with increasing particle concentration as more particles are available to stabilize smaller drops (Midmore 1999; Binks and Lumsdon 2000; Binks and Whitby 2004). However, most likely each system has a limiting drop size, which depends on the physical and mechanical properties of the system (i.e., the size of the particles and the emulsification method), and when this drop size is reached, any excess of particles will be in the continuous phase (Tcholakova et al. 2003).

**Table 5:** Different amount of OSA-modified starch mixed with oil and phosphate buffer with pH 7 and 0.2 mol/L NaCl

Starch origin	Starch added (mg/mL)	Sample ID	Emulsificationindex (6 days after vortex)	Sedimentfraction^1^ (mm^3^/mg)	Drop size (6 days after vortex)		

					*d*_32_ (μm)	*d*_43_ (μm)	CV (%)
Quinoa	50	8	0.58	0.32	270	290	32
Quinoa	100	9	0.77/0.74^2^	0.03/0.02^2^	100/110^2^	110/120^2^	37/37^2^
Quinoa	200	10	1.00	n.v.	52	55	42
Rice	50	19	0.55	1.3	550	630	41
Rice	100	20	0.55/0.62^2^	0.70/0.33^2^	530/350^2^	560/440^2^	75/63^2^
Rice	200	21	0.85	n.v.	200	310	71
Maize	50	30	0.53	0.27	1300	1400	26
Maize	100	31	0.50/0.59^2^	0.65/0.14^2^	1300/720^2^	1400/750^2^	30/29^2^
Maize	200	32	0.81	n.v.	290	300	34
Waxy barley	50	41	0.54	0.65	1200	1400	32
Waxy barley	100	42	0.58/0.60^2^	0.27/0.22^2^	690/670^2^	720/700^2^	27/27^2^
Waxy barley	200	43	0.80	n.v.	270	300	34

OSA, octenyl succinic anhydride; CV, coefficient of variation; n.v., not visible. The emulsion phase covers the bottom of the test tube and any remaining sediment in the bottom is not visible.

1 Ratio of sediment volume to added starch.

2 Replicate results from two different samples.

**Figure 12:**
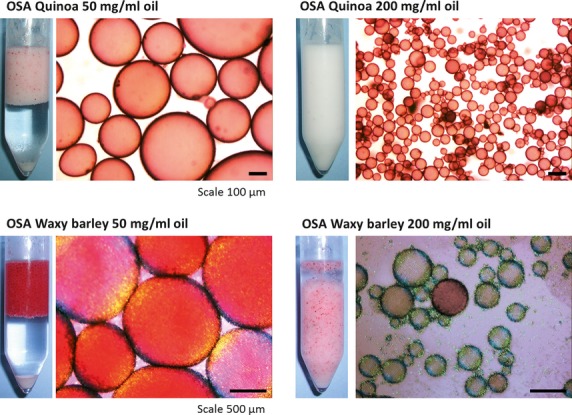
Effect of starch concentration on emulsification with octenyl succinic anhydride (OSA)-modified starch and continuous phase with 0.2 mol/L NaCl. Test tube samples and microscopic images of emulsions 6 days after vortex.

The samples with the highest amount of starch produced emulsions with a density higher than the continuous phase as shown in the sample with 200 mg starch/mL oil of waxy barley to the right in Figure 12. As the starch concentration increased, the resulting drop size decreased and the amount of starch attached to the surface of the drops increased resulting in a more stable emulsion. The volume fraction of the emulsion phase also increased as the concentration of the starch granules was increased (Table 5). Another effect of the high starch concentration was that the amount of starch granules in the water phase of the emulsion and at the drop interface increased, which is shown in Figure 12. This resulted in an increase of the total density of the drops and the emulsion phase. It is interesting to note that even at low (50 mg) starch concentrations, there was sediment of granules in the bottom of the test tubes. In fact, the starch sediment fraction decreased when the amount of starch was increased from 50 to 100 mg/mL oil. Drops formed at a lower concentration of starch granules were less covered by the granules. The emulsion was less dense at a low starch granule concentration, which means that the higher mobility of the drops and the granules promoted the sedimentation of the unabsorbed granules in the continuous phase compared with systems with a higher starch concentration, smaller drops, and a denser network.

### Starch granule size

To quantify the effects of the amount of added starch and granule size, the maximum surface coverage possible for starch concentration with a given particle size was estimated. The assumptions made were that all starch particles were identical, spherical, and attached at the oil–water interface at a contact angle of 90° with an interfacial packing fraction φ ≍0.907, that is, hexagonal close packing (Yusoff and Murray 2011). The theoretical maximum coverage, Γ_M_, was estimated using equation(7):


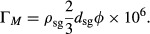
(7)

where *d*_sg_ is the surface mean diameter of the starch granule, *ρ*_sg_ is the starch density (1550 kg/m^3^), and *φ* is the packing density. Estimates of the maximum surface coverage, as well as the mean starch granule sizes for the various starches, are given in Table 6. As the surface coverage (mg/m^2^) increases with starch granule size, it is not surprising that the generated drop diameter in Figure 11 decreased with decreasing granule size as more area was covered per unit mass with smaller granules.

**Table 6:** Mean particle diameter and theoretical maximum coverage of the starch granules

Starch	*d*_10_ (μm)	*d*_32_ (μm)	*d*_43_ (μm)	Γ_M_ (mg mol/L^−2^)^1^
Native quinoa	1.14	1.7	2.51	1590
Heat quinoa	1.33	2.23	3.38	2090
OSA quinoa	1.34	2.27	3.45	2130
OSA rice	3.45	4.46	5.25	4180
OSA waxy rice	3.57	5.38	7.46	5040
OSA Hylon VII	7.07	9.32	11.1	8740
OSA waxy maize	9.54	14.7	18.0	13,800
OSA maize	11.3	14.9	17.1	14,000
OSA waxy barley	7.49	17.5	24.2	16,400

OSA, octenyl succinic anhydride; Γ_M_, theoretical maximum coverage.

1 Theoretical maximum coverage 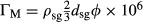 where d_sg_ is the surface mean diameter of the starch granule, ρ_sg_ is the starch density, and φ is the packing density.

The specific surface area of an emulsion, per volume of dispersed phase, is defined by


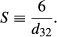
(8)

where the surface mean diameter *d*_32_ was measured by light scattering. Based on the amount of added starch, C_sg_ (as mg per mL) and the theoretical maximum coverage, Γ_M_, of the given size of starch granules, a theoretical surface area that could be generated per volume of dispersed chase can be calculated, that is,


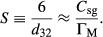
(9)

**Figure 13:**
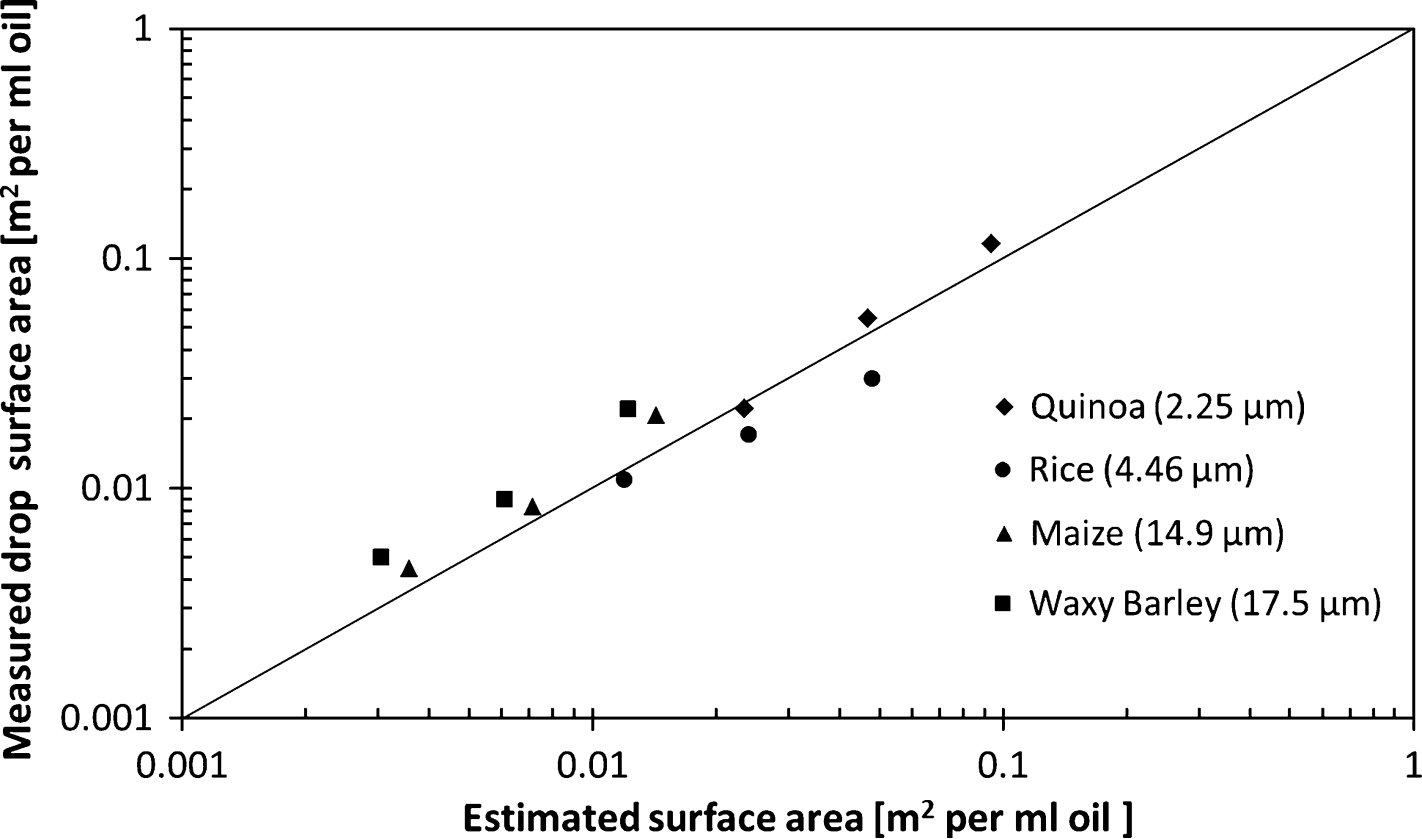
Measured specific surface area of starch-stabilized emulsions versus estimated surface area that could be stabilized for a given starch granule size and concentration. Solid represents case where the measured equals the predicted.

A comparison of the measured and calculated drop surface areas was plotted in Figure 13 and shows rather good agreement between these estimations and the measured starch stabilized drops despite the rather rough assumptions in the calculations. Starches lying above the line in Figure 13 have a larger drop area than predicted and those below the line have a smaller. A physical explanation of larger drop areas is that the assumption of hexagonal close packing overestimates the amount of starch on the surface and that it is possible to have less starch per unit area and still achieve stabilization of the drops as seen earlier in Figure 9.

By geometric analysis, it could be argued that as the ratio of starch granule size to forming drop size increases, the minimum surface coverage required to stabilize drops decreases, as larger spaces between the granules on the surface are possible while maintaining enough of a steric hinders to prevent coalescence. For this reason, the larger starch granules such as barley and maize had a larger surface area than predicted and the trend increased with increasing area (i.e., smaller drop sizes). Microscope images confirmed this, showing larger spaces and gaps on the drops surface between adsorbed starch granules. In the case of rice, it had a smaller generated area than predicted (data points that lay below the line in Fig. 13). In the microscope images of the rice emulsions, there appeared to be more free starch granules in the continuous phase and a noticeable increase in the amount of sediment. One possible explanation is that rice and maize have a less efficient surface stabilization, perhaps due to their granule morphology (Fig. 2); however, quinoa also has a significantly nonspherical granule form but also is much smaller with more rounded edges.

## Conclusions

Hydrophobic starch granules, especially when chemically modified, could be used to stabilize Pickering-type emulsions. The size of the granules had a large impact on the stabilizing capacity, and the smallest starch granules, from quinoa, had the predominantly best emulsifying properties. Also the shape of granules had slight impact on the emulsions. As expected, the emulsion droplet size decreased with increased starch concentration. The presence of salt in the continuous phase contributed to more uniform droplet sizes and less aggregation in the systems. The long-term stability of more than 2 years and the size of the emulsion drops formed make this type of starch granule-stabilized Pickering emulsions suitable for encapsulation of ingredients in food and pharmaceutical products.
